# Supplementary material for: Appraisal of the Flow Diversion Effect Provided by Braided Intracranial Stents
Source: J Clin Med. 2024 Jun 11;13(12):3409. doi: 10.3390/jcm13123409 (PMC11204822; doi:10.3390/jcm13123409)

Examples of aneurysms with recanalization/residual filling (Group A)

Stent-assisted coiling of a right middle cerebral artery aneurysm (A) was contemplated using a laser-cut stent. Due to the inability to coil the aneurysm to completion, a flow diverter was deployed after coiling (B)

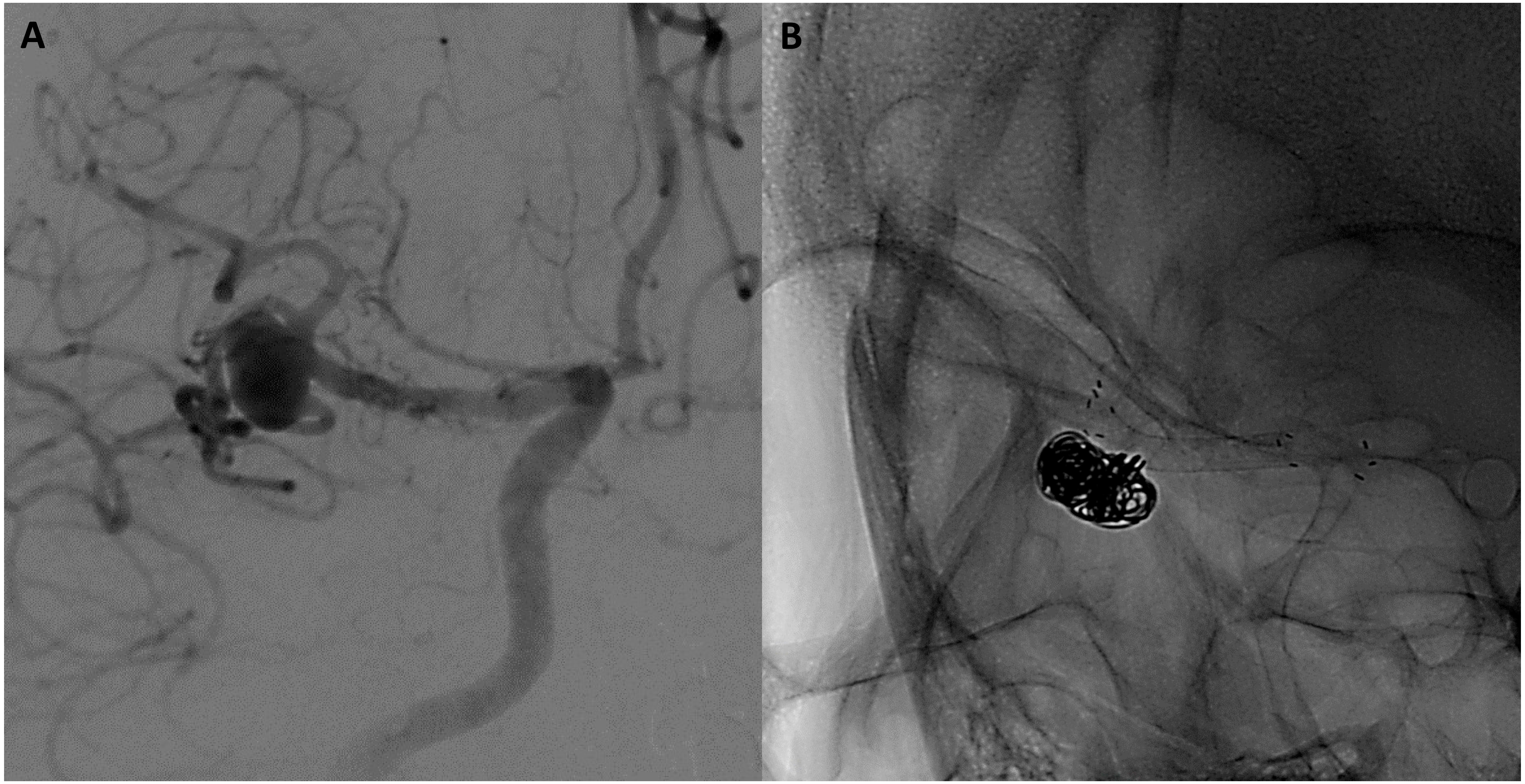

The residual neck filling (arrow, Raymond-Roy class II) on the post-procedure angiogram (A) had progressed (B) to aneurysmal dome filling (double arrows, Raymond-Roy class III) at 9-month follow-up.

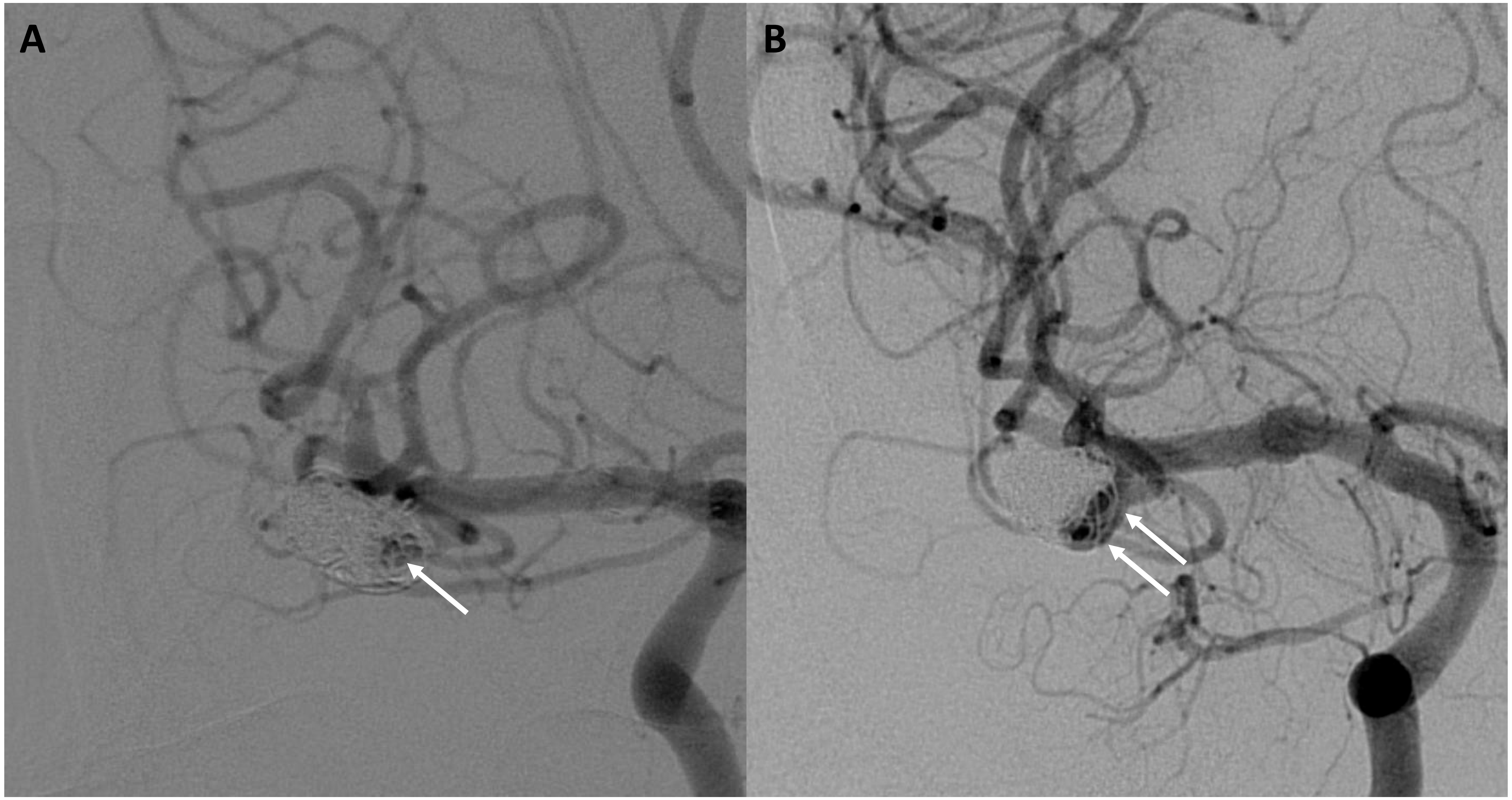

A right middle cerebral artery aneurysm (A and B) was admitted for endovascular treatment

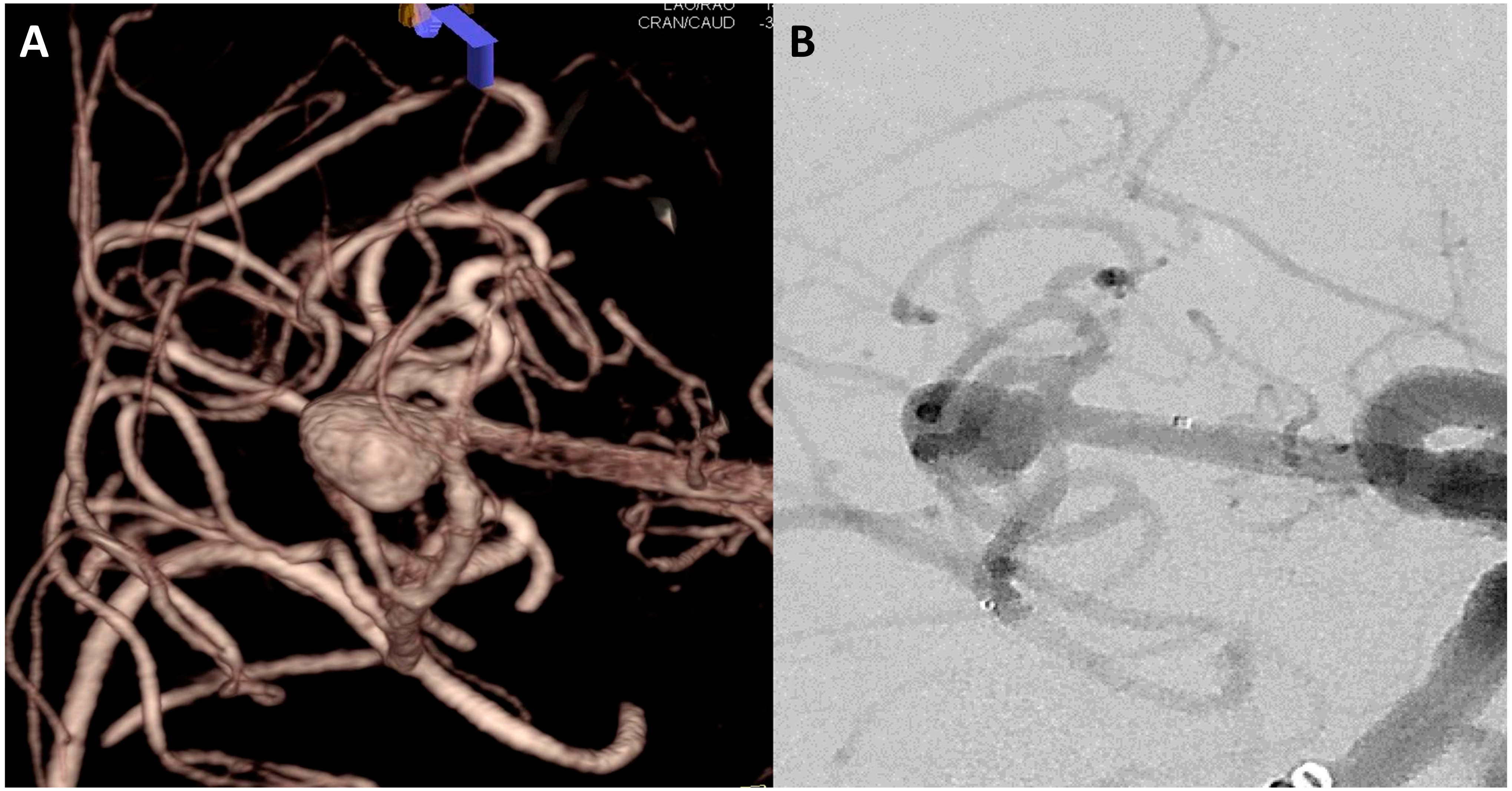

The aneurysm was coiled using a braided stent (A). A minute residual neck filling (Raymond-Roy class II) was observed on the post-procedure angiogram (B) The residual neck had progressed (C) to aneurysmal dome filling (Raymond-Roy class III) at 3-month follow-up.

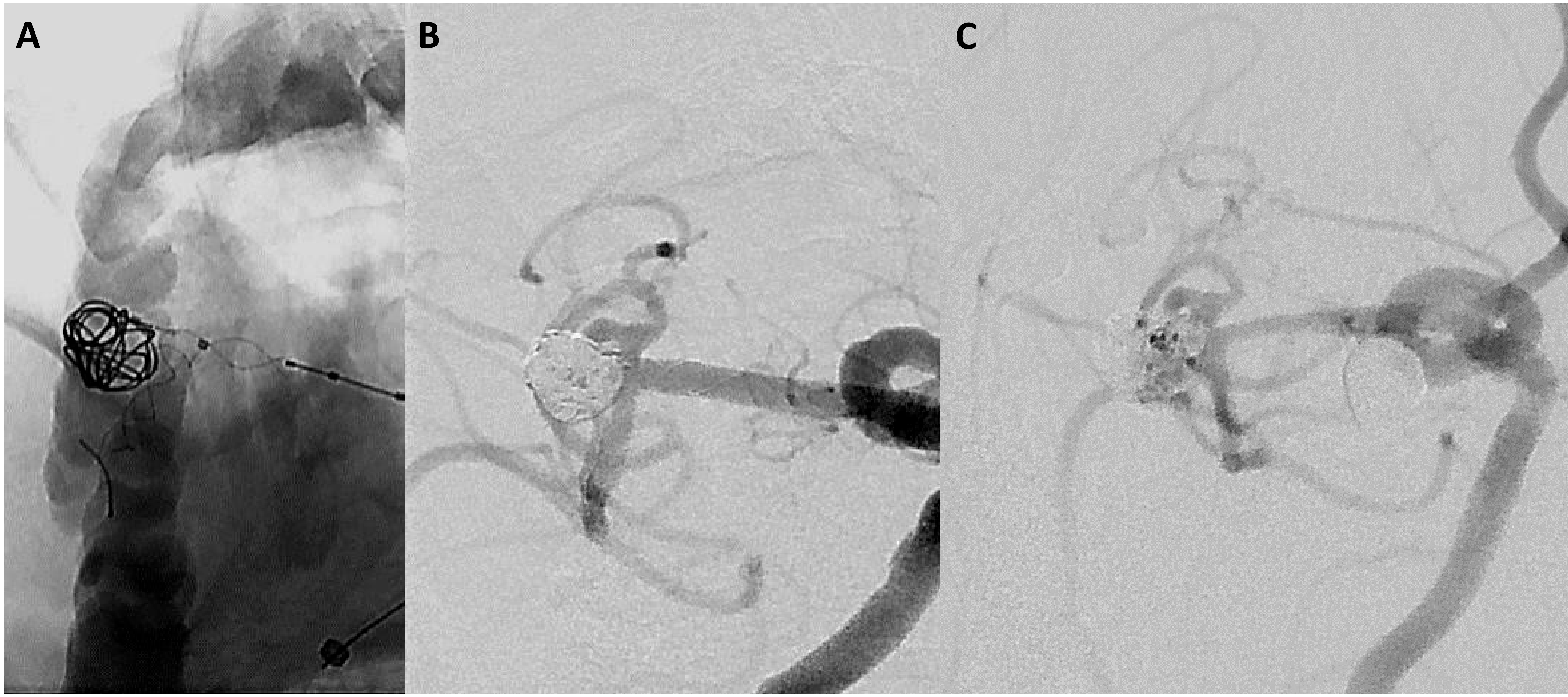

A left middle cerebral artery aneurysm (A) was treated using laser-cut stent-assisted coiling (B)

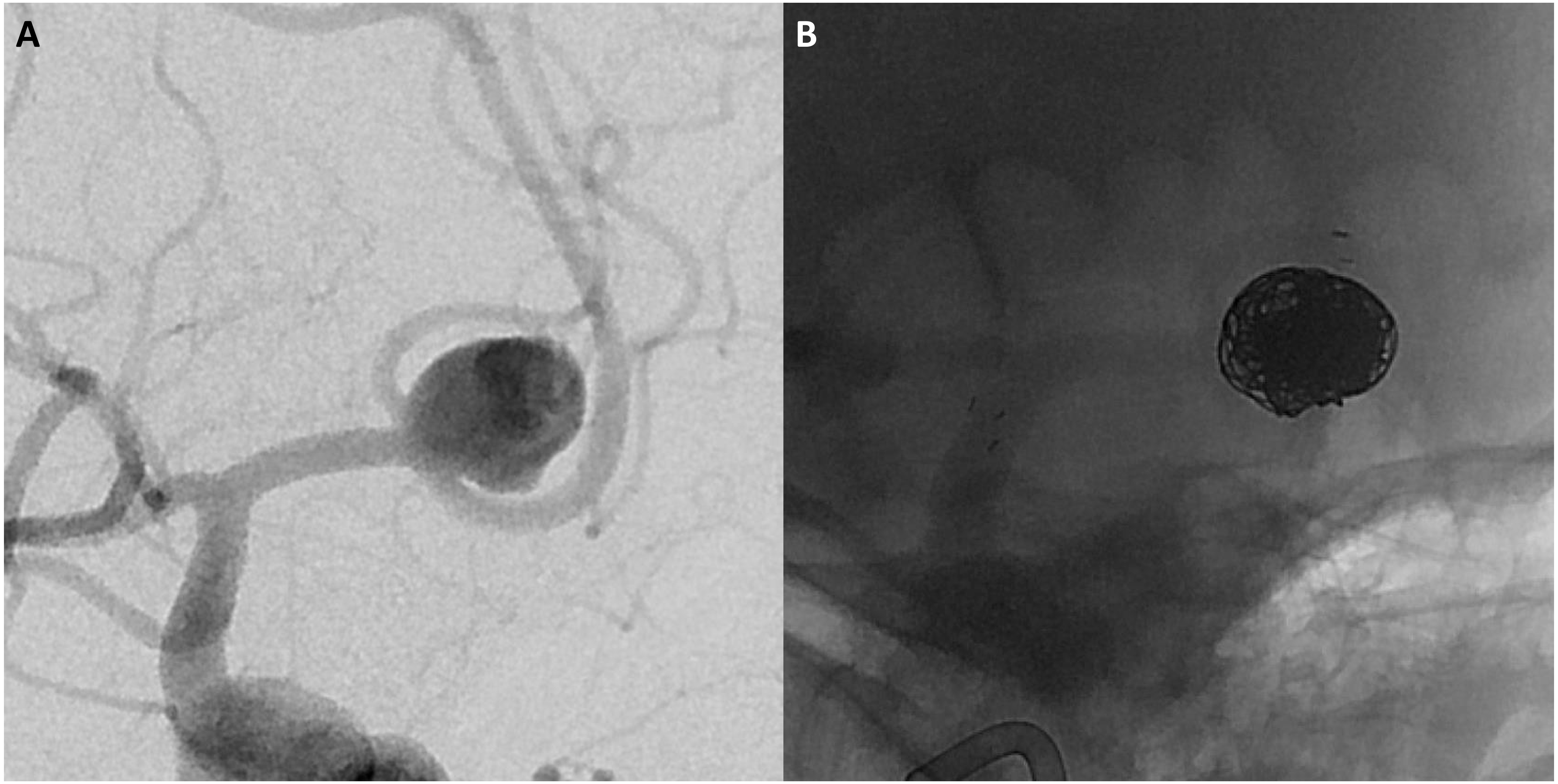

A small residual neck remnant (A, Raymond-Roy class II, arrow) was observed on the post-procedure angiogram. The residual neck had progressed (B) to aneurysmal dome filling (double arrows, Raymond-Roy class III) at 6-month follow-up.

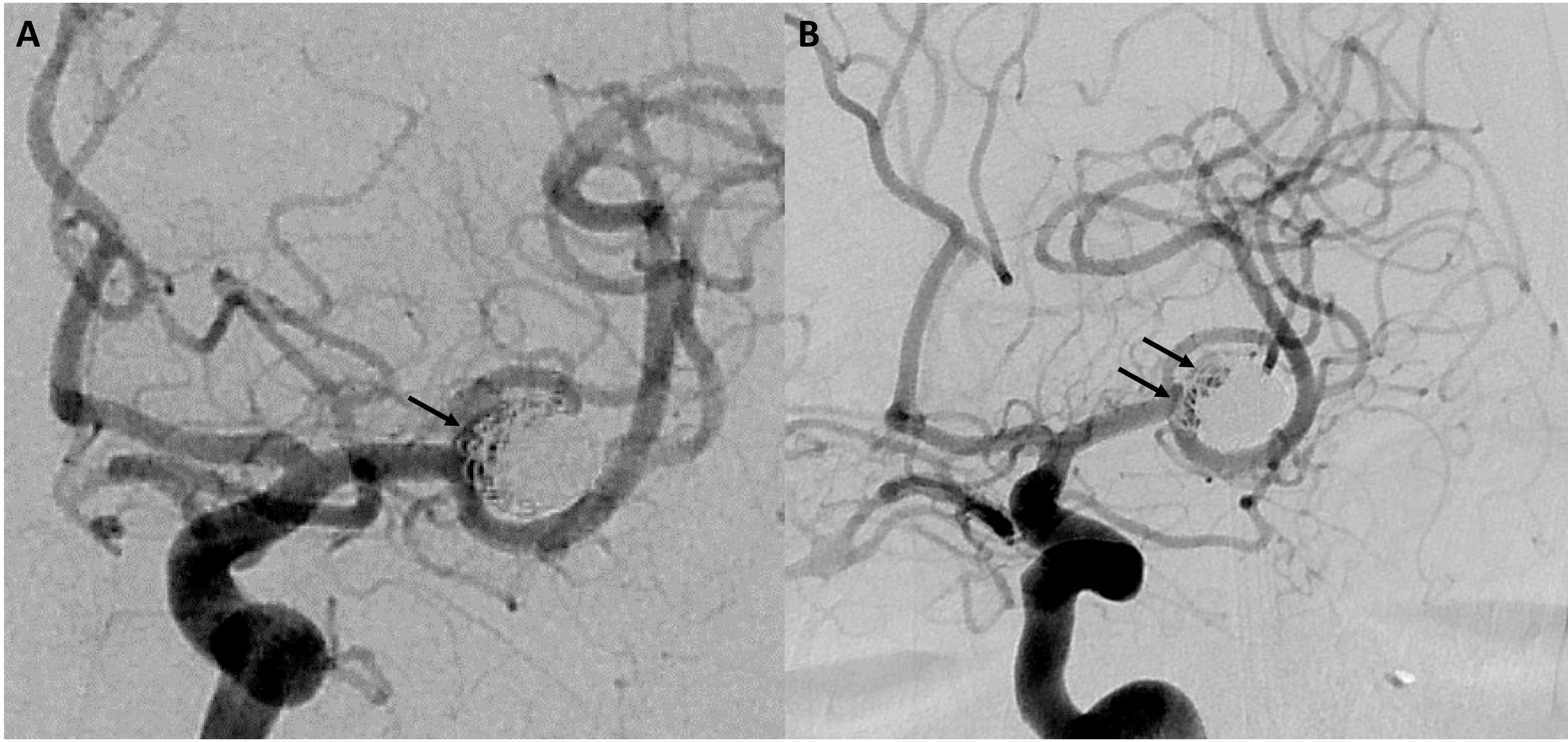

Supplement: Supplementary file 1 [file jcm-13-03409-s001.zip › Supplementary Figure S1.pdf]
