# Supplementary material for: Appraisal of the Flow Diversion Effect Provided by Braided Intracranial Stents
Source: J Clin Med. 2024 Jun 11;13(12):3409. doi: 10.3390/jcm13123409 (PMC11204822; doi:10.3390/jcm13123409)

Examples of aneurysms with stable/progressive occlusion (Group B)

A right-sided distal anterior cerebral aneurysm (A) was coiled with flow diverter assistance (B). The residual aneurysm (C) had progressed significantly at 3-month follow-up. Note the interval diminution of the callosomarginal artery (blue arrow) that arose from the aneurysm, this artery was practically invisible by 3 months.

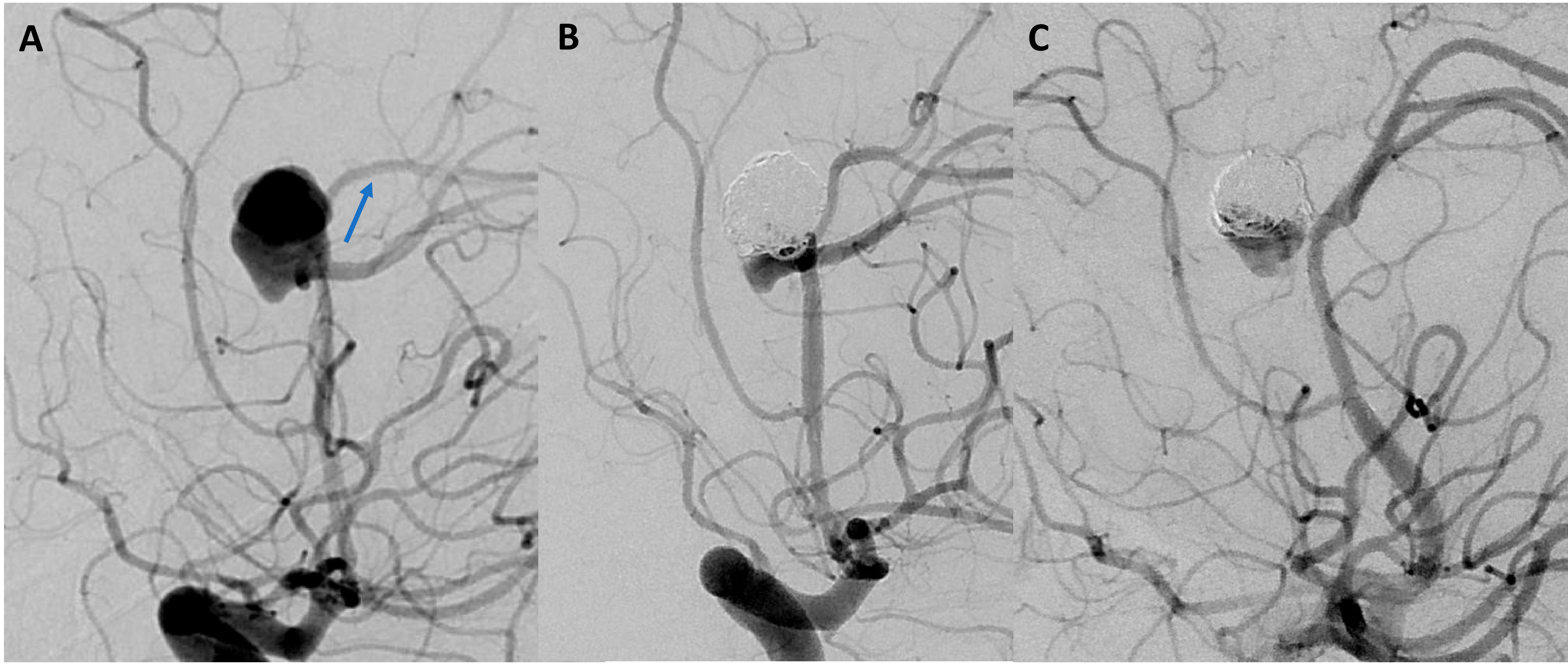

At 9 months (A and B), there is total occlusion of the aneurysm with interval occlusion of the small callosomarginal branch that was originating from the neck of the aneurysm.

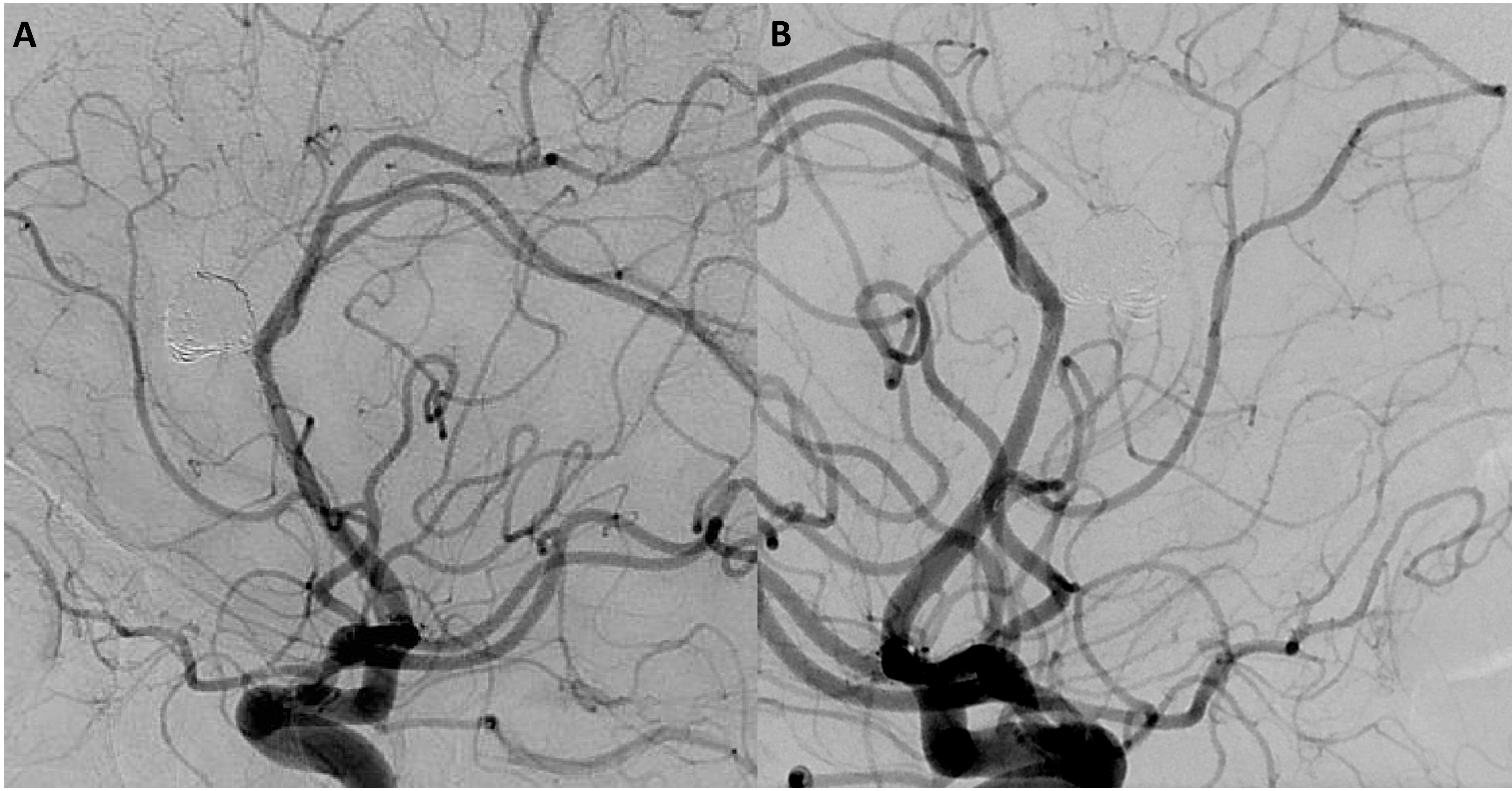

A right P1 segment aneurysm (A) was coiled using a braided stent (B). There was residual aneurysmal filling (Raymond-Roy class III) at the end of the procedure (C).

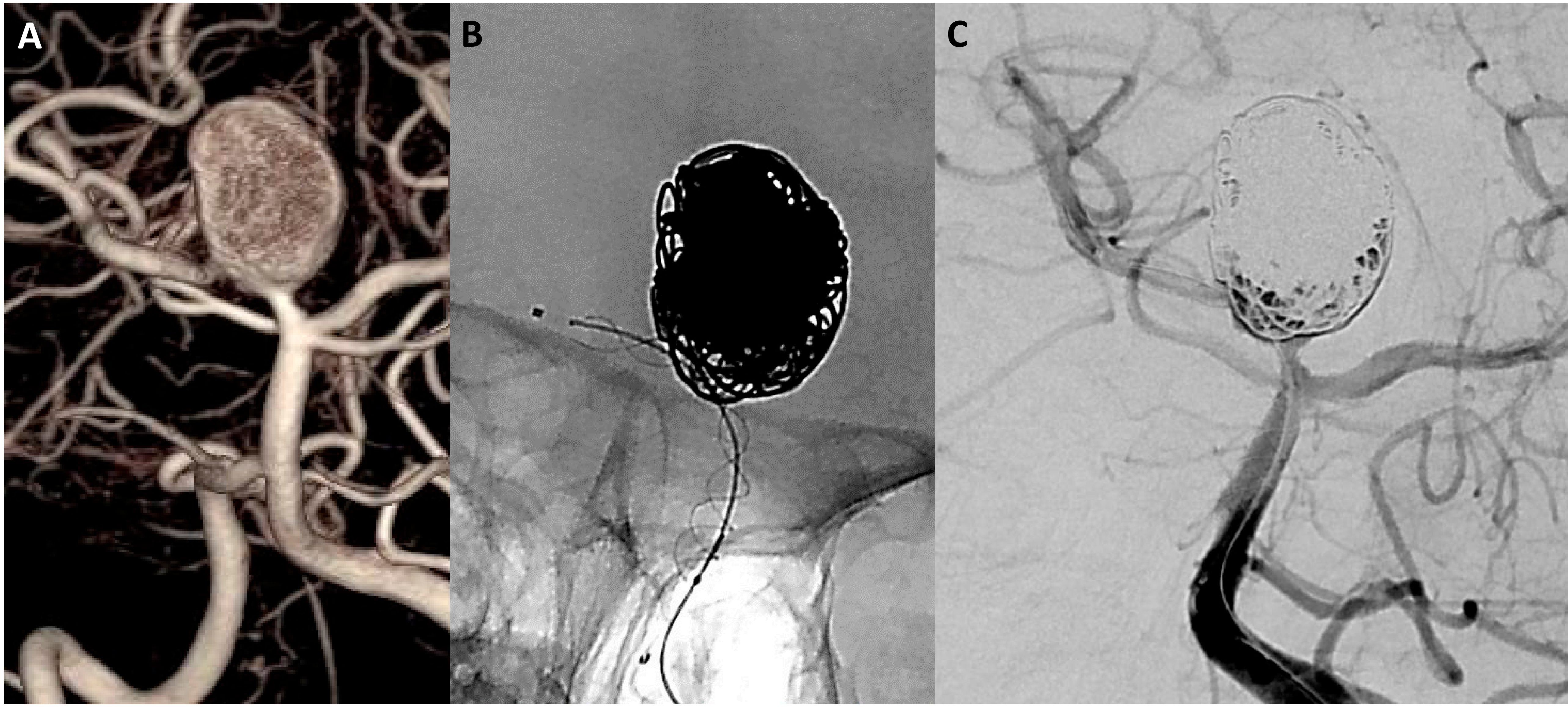

The residual aneurysm was occluded totally on the 6-month control angiogram (A and B).

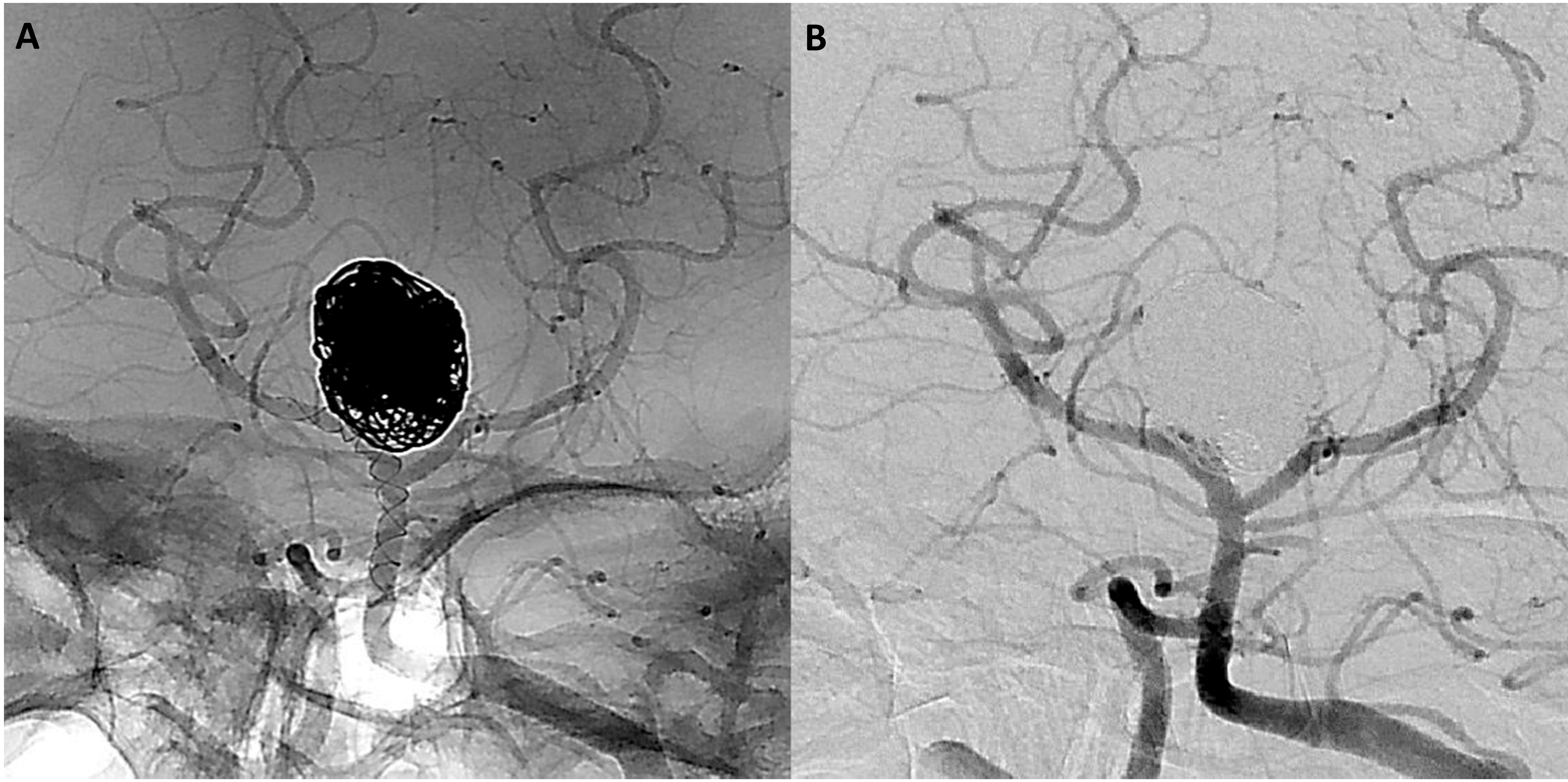

A left middle cerebral artery aneurysm (A) was treated using laser-cut stent-assisted coiling (B)

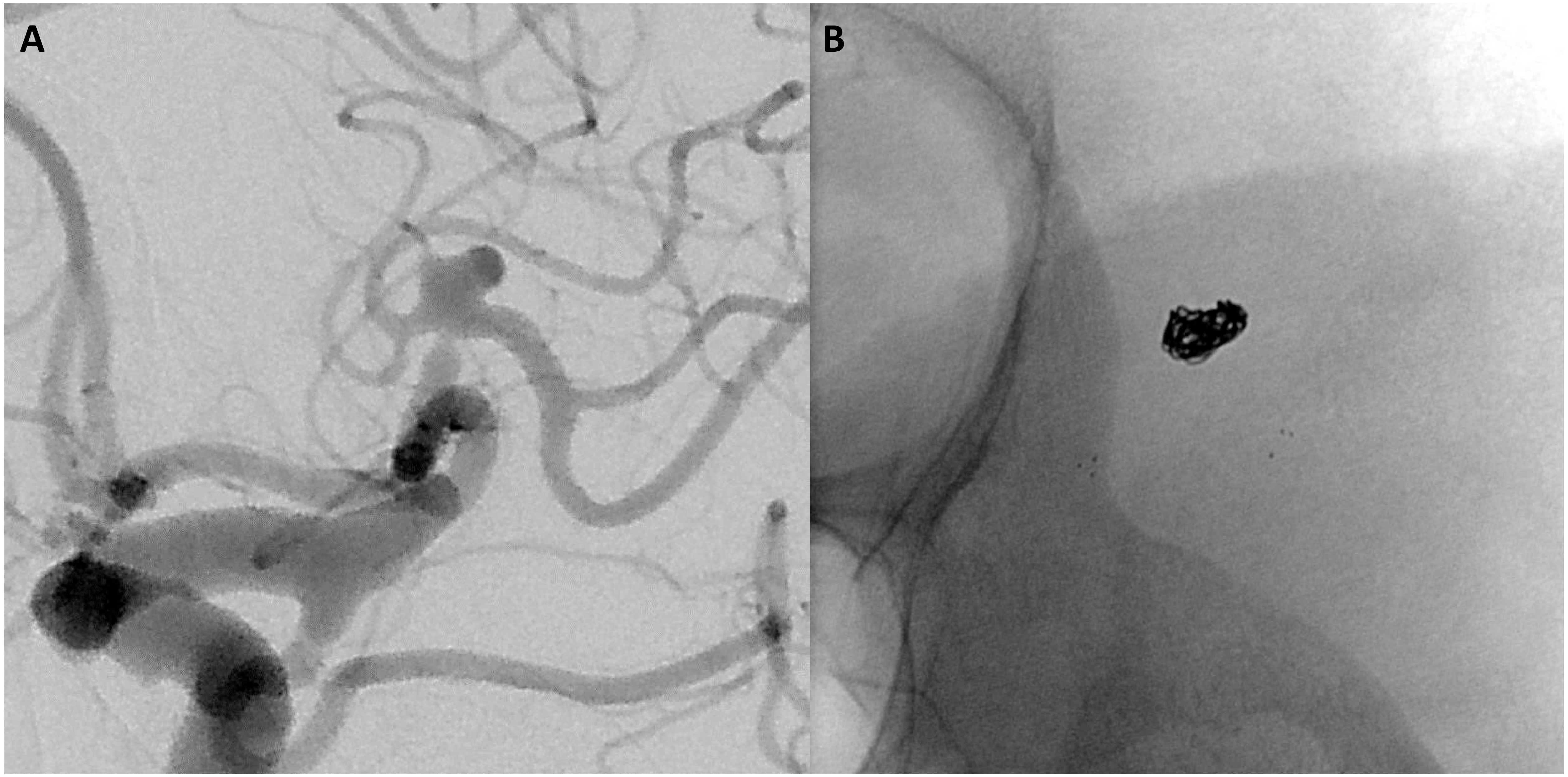

The residual aneurysm (arrows, A) was occluded totally on the 6-month control angiogram (B).

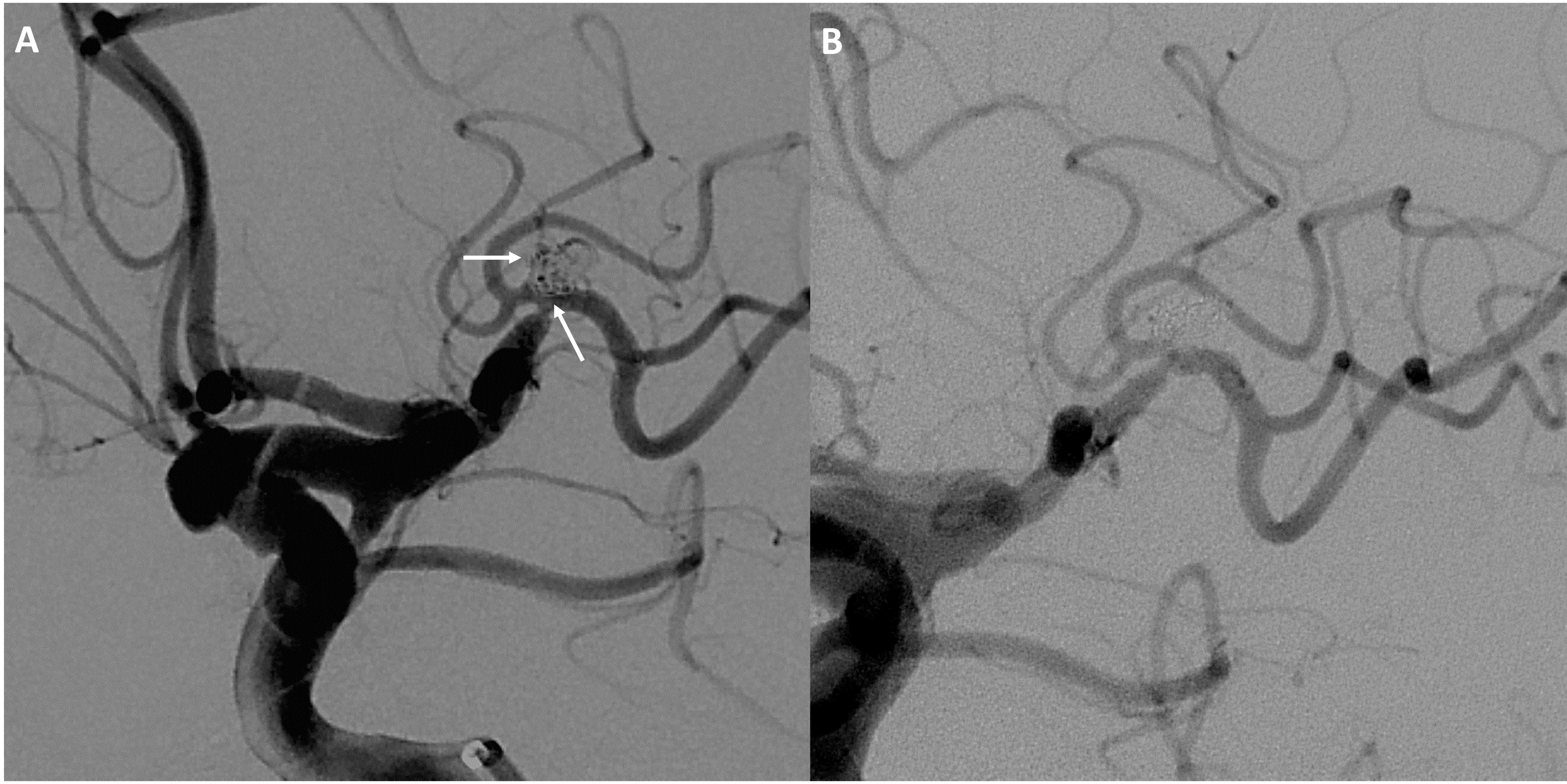

Supplement: Supplementary file 1 [file jcm-13-03409-s001.zip › Supplementary Figure S2.pdf]
