# Supplementary material for: Appraisal of the Flow Diversion Effect Provided by Braided Intracranial Stents
Source: J Clin Med. 2024 Jun 11;13(12):3409. doi: 10.3390/jcm13123409 (PMC11204822; doi:10.3390/jcm13123409)
Supplement: Supplementary file 1 [file jcm-13-03409-s001.zip › Supplementary Table S1.pdf]

**Supplementary Table S1: Types of stents used in this patient cohort**		
**Stent type**	**Stent sub-type**	**N (%)**
Braided stent (n=86)	Leo-baby	69 (35.6)
	Accero	17 (8.8)
Laser-cut stent (n=70)	Atlas (open cell)	61 (31.4)
	Acclino (closed cell)	9 (4.6)
Flow diverter stent (n=38)	Silk	13 (6.7)
	Derivo	10 (5.2)
	Derivo Mini	5 (2,6)
	Surpass Evolve	4 (2.1)
	Surpass Streamline	3 (1.5)
	Silk Mini Vista	3 (1.5)
Leo-baby, Silk, Silk Mini Vista (Balt, Montmorency, France), Accero, Acclino, Derivo, Derivo Mini (Acandis, Pforzheim, Germany). Atlas, Surpass Evolve, Surpass Streamline (Stryker, Kalamazoo, MI)		
