# Supplementary material for: Appraisal of the Flow Diversion Effect Provided by Braided Intracranial Stents
Source: J Clin Med. 2024 Jun 11;13(12):3409. doi: 10.3390/jcm13123409 (PMC11204822; doi:10.3390/jcm13123409)
Supplement: Supplementary file 1 [file jcm-13-03409-s001.zip › Supplementary Table S2.pdf]

**Supplementary Table S2: Initial RR scores according to the stent type, n (%)**			
**Stent type**	**RR1**	**RR2**	**RR3**
Laser-cut	58 (82.9)	12 (17.1)	.
Braided	64 (74.4)	19 (22.1)	3 (3.5)
Flow diverter	21 (55.3)	12 (31.6)	5 (13.2)
RR: Raymond-Roy			
