# Supplementary material for: Appraisal of the Flow Diversion Effect Provided by Braided Intracranial Stents
Source: J Clin Med. 2024 Jun 11;13(12):3409. doi: 10.3390/jcm13123409 (PMC11204822; doi:10.3390/jcm13123409)
Supplement: Supplementary file 1 [file jcm-13-03409-s001.zip › Supplementary Table S3.pdf]

**Supplementary Table S3: Aneurysms characteristics and type of device used**				
	**Laser-cut stent**	**Braided stent**	**Flow diverter**	**P value**
Aneurysm size (mm), mean±SD	6.81±3.38	7.26±3.56	9.92±4.37	<0.01
Neck width (mm), mean±SD	3.81±1.4	4.36±1.56	4.57±1.59	0.02
Proximal diameter of the stented artery (mm), mean±SD	2.52±0.57	2.58±0.44	3.07±0.93	<0.01
Distal diameter of the stented artery (mm), mean±SD	1.76±0.48	1.99±0.49	2.41±0.71	<0.01
