# Supplementary material for: Appraisal of the Flow Diversion Effect Provided by Braided Intracranial Stents
Source: J Clin Med. 2024 Jun 11;13(12):3409. doi: 10.3390/jcm13123409 (PMC11204822; doi:10.3390/jcm13123409)
Supplement: Supplementary file 1 [file jcm-13-03409-s001.zip › Supplementary Table S4.pdf]

**Supplementary Table 4: The change in RR score during the follow-up, n (%)**			
	**LCS**	**BS**	**FD**
RR3 to 1	0	2 (2.3)	4 (10.5)
RR3 to 2	0	0	0
RR3 to 3	0	1 (1.2)	1 (2.6)
RR2 to 1	3 (4.3)	14 (16.3)	10 (26.3)
RR2 to 2	6 (8.6)	5 (5.8)	2 (5.3)
RR2 to 3	3 (4.3)	0	0
RR1 to 1	51 (72.9)	61 (70.9)	20 (52.6)
RR1 to 2	7 (10)	3 (3.5)	1 (2.6)
RR1 to 3	0	0	0
Total	70 (100)	86 (100)	38 (100)
LCS: Laser-cut stent, BS: Braided stent, FD: Flow diverter, RR: Raymond-Roy			
